# An Incremental Radial Basis Function Network Based on Information Granules and Its Application

**DOI:** 10.1155/2016/3207627

**Published:** 2016-09-08

**Authors:** Myung-Won Lee, Keun-Chang Kwak

**Affiliations:** Department of Control and Instrumentation Engineering, Chosun University, 375 Seosuk-dong, Dong-gu, Gwangju 501-759, Republic of Korea

## Abstract

This paper is concerned with the design of an Incremental Radial Basis Function Network (IRBFN) by combining Linear Regression (LR) and local RBFN for the prediction of heating load and cooling load in residential buildings. Here the proposed IRBFN is designed by building a collection of information granules through Context-based Fuzzy C-Means (CFCM) clustering algorithm that is guided by the distribution of error of the linear part of the LR model. After adopting a construct of a LR as global model, refine it through local RBFN that captures remaining and more localized nonlinearities of the system to be considered. The experiments are performed on the estimation of energy performance of 768 diverse residential buildings. The experimental results revealed that the proposed IRBFN showed good performance in comparison to LR, the standard RBFN, RBFN with information granules, and Linguistic Model (LM).

## 1. Introduction

During the past few decades, we have witnessed a rapid growth in the number and variety of applications of fuzzy logic, neural networks, and evolutionary computing as a framework of computational intelligence [1–4]. We especially shall concentrate on incremental construction of Radial Basis Function Network (RBFN) with the aid of information granules. In general, we design with the simplest linear models and then refine such linear models by incorporating additional nonlinear model in system modeling. The commonly used method becomes Linear Regression (LR) model [5]. If LR model appears to be insufficient to predict, further refinements are implemented. This concept is a strong factor motivating the development of the incremental models. The effectiveness and superiority of this model have been demonstrated in the previous work. The incremental model introduced by Pedrycz and Kwak [6] represented a nonlinear and complex characteristic more effectively than conventional models. There are several advantages of this approach. First, a commonly used framework of LR has been used. The nonlinear behavior of the system could be confined to some limited regions of the input space and by adding only a few patches in these regions becomes practically relevant and conceptually justifiable. Furthermore, it has established a comprehensive design platform offering a complete set-by-step procedure of the construction of the incremental model [7]. The clustering technique used in the design of incremental model is based on Context-based Fuzzy C-Means (CFCM) clustering algorithm [8]. This clustering algorithm generates information granules in the form of fuzzy sets and estimate clusters by preserving the homogeneity of the clustered data points associated with the input and output variables. In contrast to the context-free clustering methods [9–12], context-based fuzzy clustering is performed with the use of the contexts produced in output space. The effectiveness of the CFCM clustering has been successfully demonstrated in the previous works [13–16].

In this paper, we design the variant model with the fundamental idea of incremental model. For this purpose, we design an Incremental Radial Basis Function Network (IRBFN) by incorporating LR and local RBFN for accurate quantitative prediction of energy performance of residential buildings. We adopt a design of a LR as global model and refine it through local RBFN that captures remaining and more localized nonlinearities of the system with the aid of information granulation. Here the learning methods of local RBFN are performed by LSE and Back-Propagation (BP). This research on the topic of energy performance of buildings has been recently raising concerns about energy waste. The computation of the heating and cooling load to perform the efficient building design is required to determine the specifications of the heating and cooling equipment needed to maintain comfortable indoor air conditions [17, 18]. The experiments are performed on the estimation of energy performance of 768 diverse residential buildings.

This paper is organized in the following fashion. In Section 2, the procedure steps of CFCM clustering methods are described. The entire design concept and process of IRBFN are proposed in Section 3. The experimental results are performed and discussed in Section 4. Concluding comments are covered in Section 5.

## 2. Context-Based Fuzzy C-Means Clustering

The CFCM clustering introduced by Pedrycz [8] estimates the cluster centers preserving homogeneity with the use of fuzzy granulation. First, the contexts are produced from the output variable used in the modeling problem. Next, the cluster centers are estimated by FCM clustering from input data points included in each context. By forming fuzzy clusters in input and output spaces, CFCM clustering converts numerical data into semantically meaningful information granules.

In what follows, we briefly describe the essence of CFCM clustering [8]. In a batch-mode operation, this clustering determines the cluster centers and the membership matrix by the following steps.

**Step 1 .**

Select the number of contexts *p* and cluster center *c* in each context, respectively.

**Step 2 .**

Produce the contexts in output space. These contexts were generated through a series of triangular membership functions equally spaced along the domain of an output variable. However, we may encounter a data scarcity problem due to small data included in some linguistic context. Thus, this problem brings about the difficulty to obtain clusters from the CFCM clustering. Therefore, we use probabilistic distribution of output variable to produce the flexible linguistic contexts [13].

**Step 3 .**

Once the contexts have been formed, the clustering is directed by the provided fuzzy set of each context.

**Step 4 .**

Initialize the membership matrix with random values between 0 and 1.

**Step 5 .**

Compute *c* fuzzy cluster centers using (1).

Here, fuzzification factor is generally used as fixed value *m* = 2.

(1) $$\begin{array}{c} \;v_{i}=\frac{\sum_{k=1}^{N}u_{ik}^{m}x_{k}}{\sum_{k=1}^{N}u_{ik}^{m}}. \end{array}$$

**Step 6 .**

Compute the objective function according to

(2) $$\begin{array}{c} J=\overset{c}{\underset{i=1}{\sum }}\overset{N}{\underset{k=1}{\sum }}u_{ik}^{m}d_{ik}^{2}, \end{array}$$

where *d*_*ik*_ is the Euclidean distance between *i*′th cluster center and *k*′th data point. The minimization of objective function is obtained by iteratively updating the values of the membership matrix and cluster centers. Stop if it is below a certain tolerance value.

**Step 7 .**

Compute a new membership matrix using (3). Go to Step 5.

(3) $$\begin{array}{c} u_{tik}=\frac{{w_{t}}_{k}}{\sum_{j=1}^{c}{\left(\left\|x_{k}-v_{i}\right\|/\left\|x_{k}-v_{j}\right\|\right)}^{2/\left(m-1\right)}}, \end{array}$$

where *u*_*tik*_ represents the element of the membership matrix induced by the *i*th cluster and *k*th data in the *t*th context. *w*_*t*__*k*_ denotes a membership value of the *k*th data point included by the *t*th context.

## 3. Incremental Radial Basis Function Networks (IRBFN)

**Figure 1:**
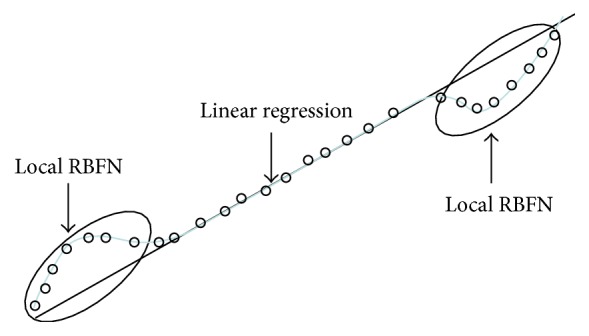
Combination of LR and local RBFN in the design of incremental RBFN (o: data points).

**Figure 2:**
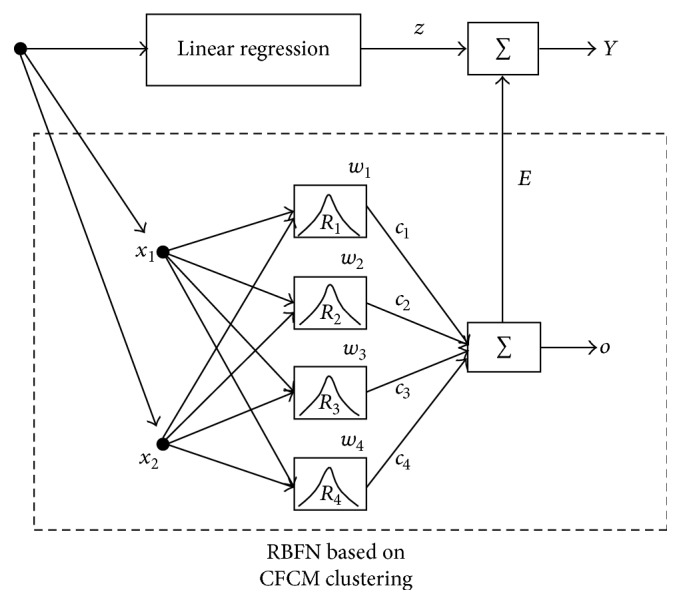
Main design process of incremental RBFN.

In this Section, we focus on two essential phases of the proposed IRBFN (Incremental RBFN) as underlying principle. First, we design a standard LR which could be treated as a preliminary construct capturing the linear part of the data. Next, the local RBFN is designed to eliminate errors produced by the regression part of the model.  Figure 1 shows the example of nonlinear relationships and their modeling through a combination of LR model of a global character and a collection of local RBFN. As shown in Figure 1, the Linear Regression exhibits a good match except for two local areas. These remaining regions are predicted by local RBFN with the use of information granules through CFCM clustering algorithm.  Figure 2 shows the architecture and overall flow of processing realized in the design of the proposed IRBFN.

The principle of the IRBFN is explained in the following steps.

**Step 1 .**

Design of Linear Regression (LR) model in the input-output space: *z* = L(**x**, **b**) with **b** denoting a vector of the regression hyperplane of the linear model, $b={\left[\begin{array}{cc} a & a_{0} \end{array}\right]}^{T}$; thus, we obtain the predicted output by using LR as a global model [6]; on the basis of the original data set, a collection of input-error pairs is formed, (**x**_*k*_, *e*_*k*_).

**Step 2 .**

Construction of the collection of contexts in the error of the regression model (*E*_1_, *E*_2_,…, *E*_*p*_): here *p* is the number of contexts; the distribution of these fuzzy sets is obtained through statistical method [7, 8] mentioned in Section 2; the contexts are characterized by triangular membership functions with a 0.5 overlap between neighboring fuzzy sets.

**Step 3 .**

CFCM clustering completed in the input-output space from the contexts produced in the error space: the obtained cluster centers are used as the centers of receptive fields in the design of local RBFN as shown in Figure 1; for *p* contexts and *c* clusters for each context, the number of nodes in hidden layer is *c* × *p*.

**Step 4 .**

Calculation of output in the design of local RBFN: the final output of RBFN is the weighted sum of the output value associated with each receptive field as follows:

(4) $$\begin{array}{c} o=\overset{c\times p}{\underset{i=1}{\sum }}w_{i}c_{i}. \end{array}$$

The receptive field functions are fixed, and then the weights of the output layer are directly estimated by LSE (Least Square Estimate) and BP (Back-Propagation). These methods are known as the most representative techniques frequently used in conjunction with RBFN [2]. In order to learn and adapt the architecture of RBFN to cope with changing environments, we need BP learning, if we use the steepest descent method to tune the centers of radial basis function and the output weights in the design of RBFN. Otherwise, we can directly obtain the output weights as one-pass estimation using LSE.

**Step 5 .**

Calculation of final output of the proposed IRBFN: the granular result of the IRBFN is combined with the output of the linear part:

(5) $$\begin{array}{c} Y=z+E. \end{array}$$

In order to evaluate the overall performance, we use standard root mean square error (RMSE) defined as follows:

(6) $$\begin{array}{c} \text{RMSE}=\sqrt{\frac{1}{N}\overset{N}{\underset{k=1}{\sum }}{\left(Y_{k}-y_{k}\right)}^{2}}. \end{array}$$

## 4. Experimental Results

In the experiments, we report on the design and performance of the proposed models to assess the heating load and cooling load requirements of building as a function of building parameters. All experiments were completed in the 10-fold cross-validation mode with a typical 60%–40% split between the training and testing data subsets. We perform energy analysis using 12 different building shapes simulated in Ecotect [17, 18].

These 12 building forms were generated by taking the elementary cube (3.5 × 3.5 × 3.5) where each building form is composed of 18 elements. The materials used for each elementary cube are the same for all building forms. The selection was made by the newest and most common materials in the building construction industry and by the lowest *U*-value [17]. The buildings differ with respect to the glazing area, the glazing area distribution, orientation, overall height, roof area, wall area, surface area, and relative compactness. The data set comprises 768 samples and 8 features. The attributes to be predicted in terms of the preceding 8 input attributes are two real valued responses (heating load and cooling load).

**Figure 3:**
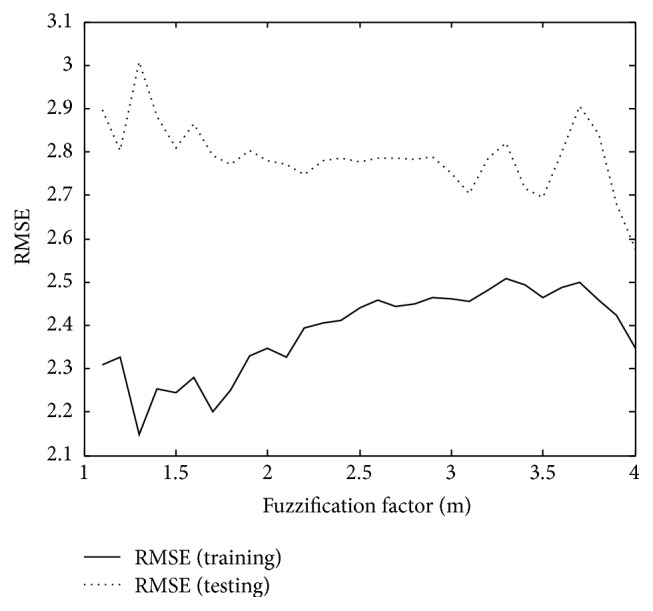
RMSE variation by the values of the fuzzification coefficient (*p* = *c* = 6).

We obtained the experimental results with the two essential parameters (*p*, *c*) controlling the granularity of the construct in the input and output space. The numerical range of the fuzzification factor (*m*) used in the experiments is between 1.5 and 3.0 with the incremental step of 0.1.  Table 1 listed the optimal values of the fuzzification factor by the increase of the number of contexts and clusters.  Figure 3 shows the variation of the RMSE caused by the fuzzification factor in the case of *p* = *c* = 6 for heating load prediction. Here the optimal values of the parameters are such that the testing error becomes minimal.

**Figure 4:**
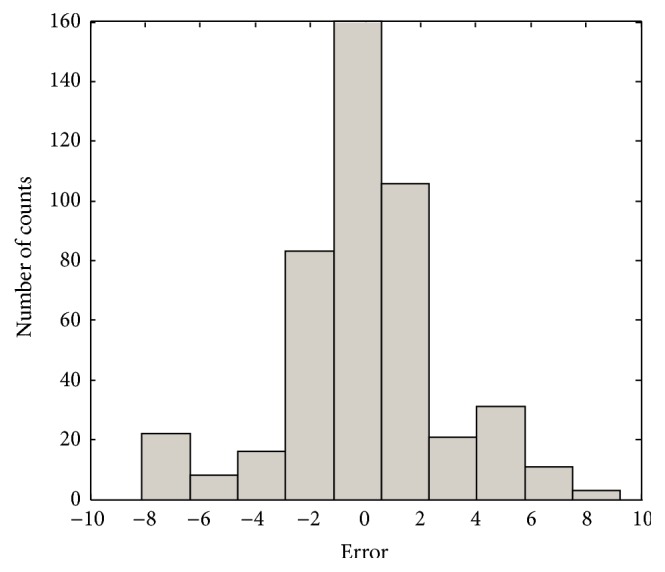
Histogram in the error space.

**Figure 5:**
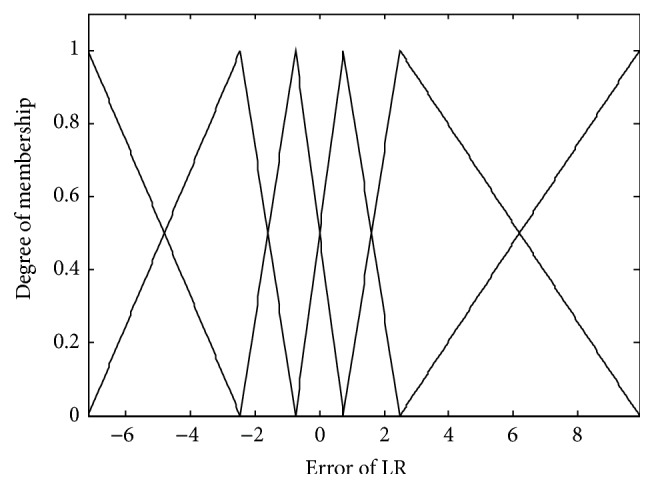
Contexts generated in the error space.

**Figure 6:**
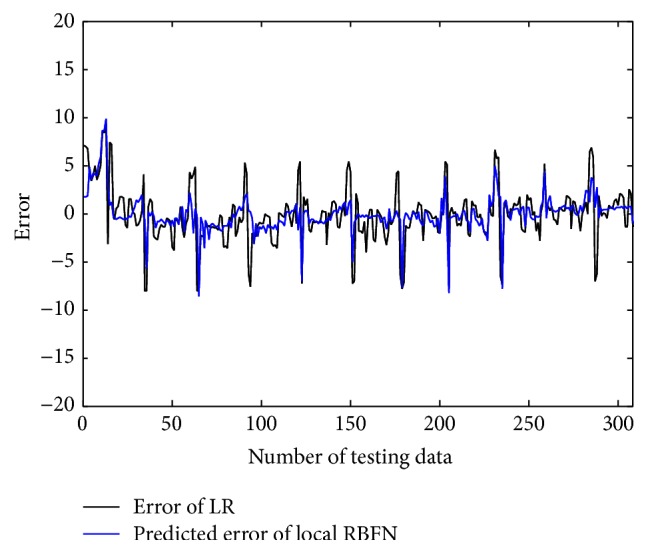
Prediction by local RBFN for the error of LR.

**Figure 7:**
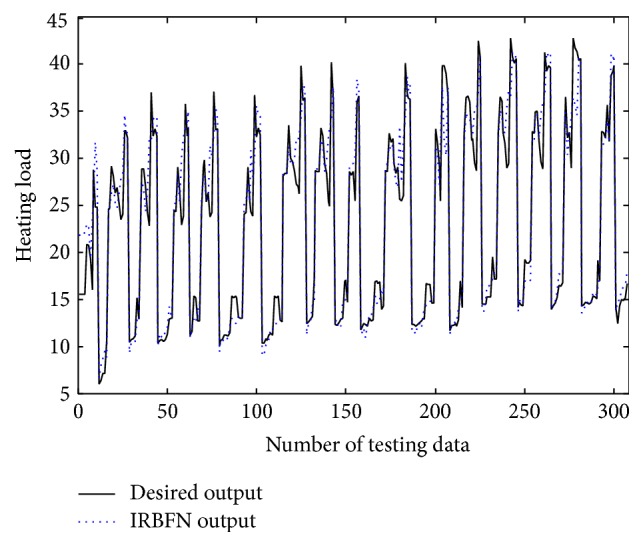
Prediction performance for heating load.

**Figure 8:**
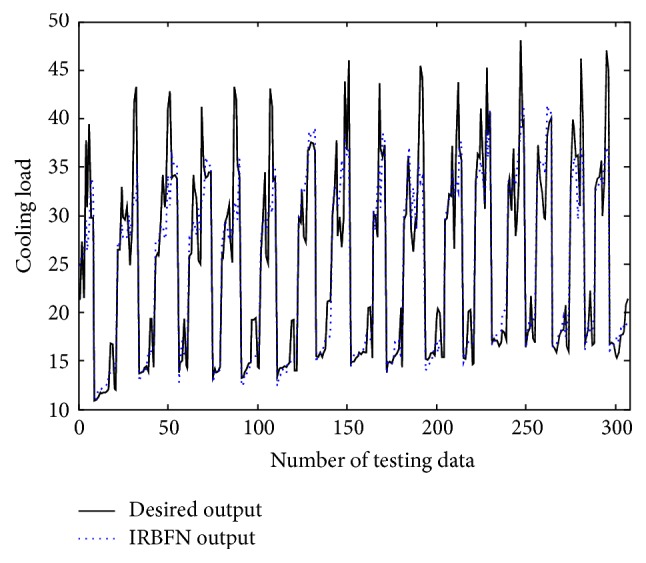
Prediction performance for cooling load.

In the conventional method [14], the contexts were produced through triangular membership functions, equally spaced along the domain of an output variable. However, we may encounter a data scarcity problem due to small amounts of data included in some context. Thus, we use a probabilistic distribution of the output variable to obtain flexible contexts. For this, the contexts in the error space are produced based on a histogram shown in Figure 4, Probability Density Function (PDF), and Conditional Density Function (CDF) [13].  Figure 5 shows the contexts (*p* = 6) generated in the error space of LR model as one example among 10-fold cross-validation mode.  Figure 6 shows the prediction performance based on local RBFN for the error of LR. As shown in Figure 6, the result clearly shows that the local RBFN with the use of information granulation has good prediction capability. Figures 7 and 8 show the performance of IRBFN based on LSE for the prediction of heating load and cooling load, respectively. Here the number of epochs is 1000 and learning rate is 0.01, respectively. As shown in these figures, the proposed IRBFN showed good generalization capability for testing data set, respectively. Tables 2 and 3 listed the comparison results of RMSE for the prediction of heating and cooling load, respectively. As listed in these tables, the experimental results revealed that the proposed IRBFN showed good performance in comparison to LR, MLP (Multilayer Perceptron), the conventional RBFN, RBFN with CFCM clustering, and LM (Linguistic Model).

## 5. Conclusions

We developed the incremental RBFN by combining LR and local RBFN for the prediction of heating load and cooling load of residential buildings. It was found from the result that the proposed IRBFN has good approximation and generalization capabilities with the aid of information granulation. These results lead us to the conclusion that the proposed IRBFN combined by LR and local RBFN showed a good performance in comparison to the previous works. For further research, we shall design this model to optimize the number of contexts and clusters per context based on evolutionary algorithm.

## Figures and Tables

**Table 1:** Optimal values of the fuzzification coefficient for selected number of contexts (*p*) and clusters (*c*).

*p*	*c*			
	3	4	5	6
3	2.6	2.1	2.2	2.3
4	2.2	2.1	2.2	2
5	1.9	1.9	1.9	2
6	1.7	1.8	1.8	2

**Table 2:** Comparison results of RMSE for the prediction of heating load (mean value of 10-fold cross-validation) (*∗*: number of rules).

Methods	Number of nodes	RMSE (training)	RMSE (testing)
LR	—	2.936	2.911
MLP	36	2.883	2.890
RBFN	36	3.707	5.199
RBFN (CFCM) [14]	36	2.767	3.106
LM	36^*∗*^	4.084	4.388
Proposed incremental method			
IRBFN_LSE	36	2.284	2.826
IRBFN_BP	36	2.353	2.730

**Table 3:** Comparison results of RMSE for the prediction of cooling load (mean value of 10-fold cross-validation) (*∗*: number of rules).

Methods	Number of nodes	RMSE (training)	RMSE (testing)
LR	—	3.180	3.208
MLP	36	3.176	3.226
RBFN	36	3.601	4.812
RBFN (CFCM) [14]	36	2.866	3.388
LM	36^*∗*^	3.866	4.296
Proposed incremental method			
IRBFN_LSE	36	2.462	3.102
IRBFN_BP	36	2.555	3.089
